# Ambient versus household PM_2.5_ exposure and socioeconomic disparities in intracerebral hemorrhage burden: a 32-year global analysis (1990–2021) with projections to 2050

**DOI:** 10.3389/fpubh.2025.1615934

**Published:** 2025-06-18

**Authors:** Erman Wu, Riqing Su, Tong Tang, Guohua Zhu, Dangmurenjiafu Geng

**Affiliations:** ^1^Department of Neurosurgery, The First Affiliated Hospital of Xinjiang Medical University, Ürümqi, China; ^2^Department of Computer Science and Information Technologies, Elviña Campus, University of A Coruña, A Coruña, Spain

**Keywords:** PM_2.5_, intracerebral hemorrhage, health disparities, GBD 2021, temporal trends

## Abstract

**Background:**

While hypertension dominates intracerebral hemorrhage (ICH) risk globally, PM_2.5_ exacerbates health inequities through distinct ambient (APMP) and household (HAP) exposure pathways. Quantifying PM_2.5_-attributable burden across socioeconomic gradients remains critical for targeted intervention.

**Methods:**

Using Global Burden of Disease (GBD) 2021 data, we analyzed age-standardized mortality (ASMR), disability-adjusted life years (DALYs), years lived with disability (YLDs), and years of life lost (YLLs) for PM_2.5_-attributable ICH. Joinpoint regression assessed trends (AAPC), while Bayesian Age-Period-Cohort modeling projected burdens to 2050. Pollution sources (APMP/HAP) were stratified by Socio-demographic Index (SDI).

**Results:**

Globally, PM_2.5_ caused 995,650 ICH - related deaths and 24,015,340 DALYs in 2021. From 1990 to 2021, the ASMR and disability rates for ICH due to PM_2.5_ exposure showed consistent declines globally (ASMR: −52.4%, DALYs: −53.1%, YLL: −53.4%, YLD: −40.7%), driven by HAP reductions. However, the absolute number of deaths and YLDs rose. The AAPC of the PM_2.5_ - related ICH burden also declined in the past 30 years. Nevertheless, projections indicate that by 2050, the PM_2.5_ - related ICH burden will increase. Low SDI regions exhibited 24.7 times higher ASMR than those in high SDI areas. Regionally, Asian regions (East/South/Southeast Asia) had the highest death counts of ICH due to PM_2.5_. APMP dominated in High SDI regions (e.g., Western Europe, North America, and Australasia), whereas HAP remained prevalent in low-SDI settings (e.g., Sub-Saharan Africa). Mortality disparities extended to demographics, with males experiencing 1.8 times higher ASMR than females, and the peak of fatalities shifting to older age groups (from 65–69 to 70–74 years). A strong inverse correlation emerged between SDI and the burden (ASMR-SDI: *r* = −0.76, *p* < 0.001).

**Conclusion:**

Analysis of GBD 2021 data reveals PM_2.5_-attributable ICH mortality in Low SDI regions is 24.7 times higher than High SDI areas, driven by HAP vs. APMP. Despite declining age-standardized rates globally (1990–2021), absolute DALYs and YLDs rose. Projections indicate burden resurgence by 2050. Considering population aging, gender and regional disparities (Asia and Sub-Saharan Africa bears highest burden), it is urgent to develop targeted strategies for APMP and HAP.

## Introduction

Intracerebral hemorrhage (ICH) is a type of stroke characterized by the accumulation of blood within the brain parenchyma, often resulting from the rupture of a blood vessel (1). ICH accounting for 28.8% of global stroke cases in 2021, which affect 3.4 million people and cause 79.5 million disability-adjusted life years lost (2). The etiological spectrum of ICH includes various risk factors, hypertension, cerebral amyloid angiopathy, and certain coagulopathies, all of which contribute to the fragility of cerebral vasculature leading to rupture and subsequent bleeding (3–5). Unlike ischemic stroke, ICH pathophysiology involves abrupt hematoma expansion and secondary injury mechanisms (e.g., cerebral edema, neuroinflammation), contributing to its disproportionately poor prognosis (6, 7). ICH is associated with high morbidity and mortality. In - hospital mortality rates range from 27.1 to 37.5%, and the 2 - year mortality reaches up to 49.5%. Only 14.5% of symptomatic ICH patients can be discharged home independently, while over 34% are transferred to long - term nursing care facilities (8).

Fine particulate matter (PM_2.5_) is a global public health concern, which link to many non-communicate diseases including cardiovascular disease (CVD), COPD, lung cancer, chronic kidney disease, and stroke [Schraufnagel et al. (9)]. Moreover, it contributed to 21% of stroke death and 19% CVD death. The mechanisms by which PM_2.5_ impacts human health primarily involve triggering oxidative stress, prompting the secretion of cytokines, causing damage to DNA, modifying gene expression patterns, inducing immune - related toxicity, provoking inflammatory reactions, and initiating apoptosis (10). Emerging evidence implicates particulate matter pollution, particularly PM_2.5,_ as a modifiable risk factor for ICH. PM_2.5_ exposure induces systemic vascular dysfunction through dual pathways: (1) pro-inflammatory cytokine release and endothelial activation, increasing blood–brain barrier permeability (11, 12); and (2) oxidative stress-mediated impairment of nitric oxide signaling, elevating systolic blood pressure — a key driver of cerebral small vessel rupture (13, 14). Epidemiological studies further demonstrate that acute PM_2.5_ exposure (per 10 μg/m^3^ increase) elevates ICH risk by 12–15%, with synergistic effects observed in the presence of nitrogen dioxide (NO₂) (15, 16). Critically, these effects exhibit subtype specificity: while PM_2.5_ robustly correlates with ICH incidence (OR = 1.24, 95% CI: 1.12–1.38), no significant association exists for subarachnoid hemorrhage, underscoring distinct pathological mechanisms (17).

Despite a marginal global decline in PM_2.5_ exposure (−0.2% annual average reduction from 2000 to 2019), 65% of urban areas experienced increasing concentrations. Improvements occurred in the eastern United States, Europe, southeastern China, and Japan, whereas the worsening occurred in the Middle East, sub - Saharan Africa, and South Asia (18). Merely 5% of the global land area – predominantly remote regions of North America and Asia– maintains annual mean concentrations below 5 μg/m^3^, whereas over 90% of the human population endures exposure exceeding WHO’s safety threshold (19). The Global Burden of Disease (GBD) 2021 study revealed that 2.67 billion individuals (33.8% of the global population) were exposed to household air pollution (HAP) at a mean concentration of 84.2 μg/m^3^. While this represents a significant reduction from the 56.7% exposure rate observed in 1990, the absolute number of exposed individuals decreased by only 0.35 billion (10%). Despite the notable decline in HAP exposure prevalence, a substantial proportion of the global population remains affected, with marked regional and gender disparities. Population growth has offset the health gains achieved through reduced exposure levels, particularly in high-burden regions. Targeted interventions are imperative, with a specific focus on vulnerable populations and geographical areas (20).

Xu et al. (21) investigated the disease burden of ICH and its attributable risk factors from 1990 to 2021 using GBD 2021 data. Their study examined relevant risk factors, it did not conduct an in-depth analysis of temporal trends in particulate matter pollution (PMP) subtypes, mortality rates associated with PMP exposure, impact on years lived with disability (YLDs) and years of life lost (YLLs), age- and gender-specific variations in PMP-related ICH outcomes.

To address these critical gaps, our study leverages GBD 2021 data, this study addresses these gaps by analyzing 30-year spatiotemporal trends in PM_2.5_-attributable ICH mortality and disability across 204 nations. Our findings aim to inform targeted interventions aligned with Sustainable Development Goals (SDGs), prioritizing populations at highest risk.

## Methods

### Data sources

The GBD 2021 Dataset v2.0, released in 2024 by the GBD Collaborative Consortium, provided epidemiological estimates for 371 diseases and injuries across 204 countries/territories from 1990 to 2021 (22, 23). This study extracted data on PM_2.5_-attributable ICH burden—including incidence, mortality, DALYs, YLDs, and YLLs—from the Global Health Data Exchange (GHDx) platform.^1^ Data were stratified by: Demographics: Sex and 5-year age groups (0–4 to ≥95 years). Socioeconomic status: Socio-demographic Index (SDI) quintiles (low to high). Geography: National and subnational levels (21 GBD regions). Uncertainty intervals (95% UIs) for all estimates were derived through ensemble modeling, accounting for input data variance and model specification heterogeneity. Detailed methodologies for GBD 2021 data acquisition, statistical modeling, and uncertainty quantification are described in prior publications.

### Definition

PM_2.5_ was divided into ambient Particulate Matter Pollution (APMP) and household air pollution (HAP) in GBD 2021. APMP primarily manifests as the annual average concentration of fine particulate matter with a diameter of less than 2.5 micrometers in the air, weighted by population exposure levels. The assessment integrates data from multiple sources, including satellite remote sensing of atmospheric aerosol parameters, real-time data collected by ground-level air quality monitoring equipment, computational results from atmospheric chemical transport models, as well as basic information on population distribution and land use. In the GBD 2021 study, researchers expanded the existing database by not only updating observation records from existing monitoring stations but also adding new data (24). HAP refers to PM_2.5_ exposure resulting from the combustion of solid fuels (including wood, coal/charcoal, animal dung, and crop residues). The exposure assessment is calculated by multiplying the prevalence of solid fuel dependency by corresponding PM_2.5_ exposure levels. For detailed methodology, please refer to the GBD 2021 HAP Collaborators’ study published in previous article (24, 25).

The International Classification of Diseases, 10th Revision (ICD-10) classifies intracerebral hemorrhage under codes I61-I62 (including subcategories I62.1-I62.9), I68.1-I68.2, and I69.1-I69.2. Corresponding ICD-9 codes include 431–432.9 and 437.2 (26).

Age-standardized rates (ASRs) are expressed per 100,000 individuals, utilizing a standardized age distribution to enable equitable comparisons across regions. Disability-adjusted life years (DALYs) serve as a metric for disease burden, combining years of life lost (YLL) and years lived with disability (YLD), as defined in prior publications.

The SDI quantifies countries and territories‘s developmental status, integrating standardized indicators of total fertility rate, educational attainment, and per capita income. This index enables comparative socioeconomic analyses across geographically and politically distinct regions. SDI values are scaled from 0 (lowest development) to 100 (highest development). 204 countries and territories were stratified into five development quintiles (categorized as low, low-middle, middle, middle-high, and high SDI) and subsequently aggregated into 21 GBD regions for hierarchical modeling (2, 25, 27).

### Statistical analysis

The study compared the death, DALYs, YLL and YLDs between the sexes, age, SDI (five categories), regions (21 GBD regions), and countries (204 countries and territories). The temporal trend was evaluated using the Joinpoint Regression Program (Version 5.0.2), and the average annual percent change (AAPC) was calculated during 1990–2021 with default parameters. To investigate the factors influencing PAP, the association between ASRs and SDI, it was assessed at the national level using generalized linear model (GLM) and the Pearson test was used to determine statistical significance. Statistical analyses and the visualization of results were conducted using the R software (version 4.3.1, R Core Team).

### Projection analysis

The analysis was conducted using the Bayesian Age-Period-Cohort (BAPC) framework implemented in R to project future disease epidemiology (18). The principle of BAPC is based on the Bayesian statistical framework. By constructing a hierarchical model, it decouples the age effect, period effect, and cohort effect in the time - series data. Then, it uses the Markov Chain Monte Carlo method for parameter estimation to quantify uncertainty and integrate prior knowledge to achieve dynamic prediction. Mortality patterns were examined through a dual-data approach incorporating observed age-stratified population statistics (1980–2021); Forecasted demographic distributions (2022–2050). To overcome the characteristic identifiability challenges in APC modeling, the framework incorporated: Constraint-based parameter estimation; Flexible smoothing via quintic B-spline basis functions for age and temporal dimensions; Hierarchical spatial priors (iGMRF) to maintain neighborhood structure among consecutive age/period parameters. Bayesian posterior distributions were approximated using MCMC simulation techniques. Model reliability was quantified through iterative validation procedures excluding individual data points sequentially.

## Results

### Global burden patterns and temporal trends of PM_2.5_-attributable intracerebral hemorrhage (1990–2021) and future projection to 2050

**Figure 1:**
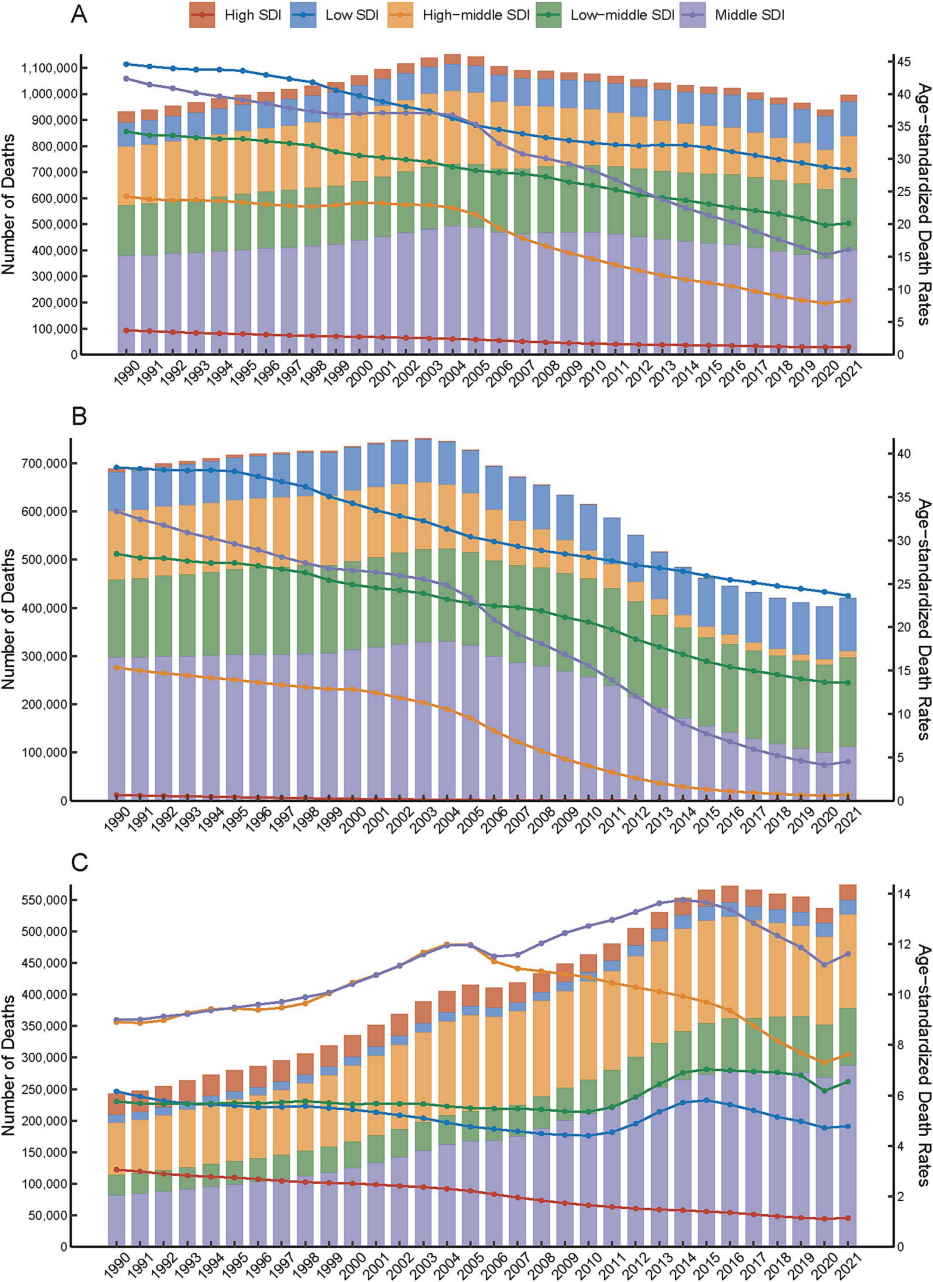
Temporal trends in the number and age-standardized rates of PM_2.5_-attributable intracerebral hemorrhage (ICH) burden across Socio-demographic Index (SDI) quintiles, 1990–2021. Total PM_2.5_ **(A)**, HAP **(B)**, APMP **(C)**.

**Figure 2:**
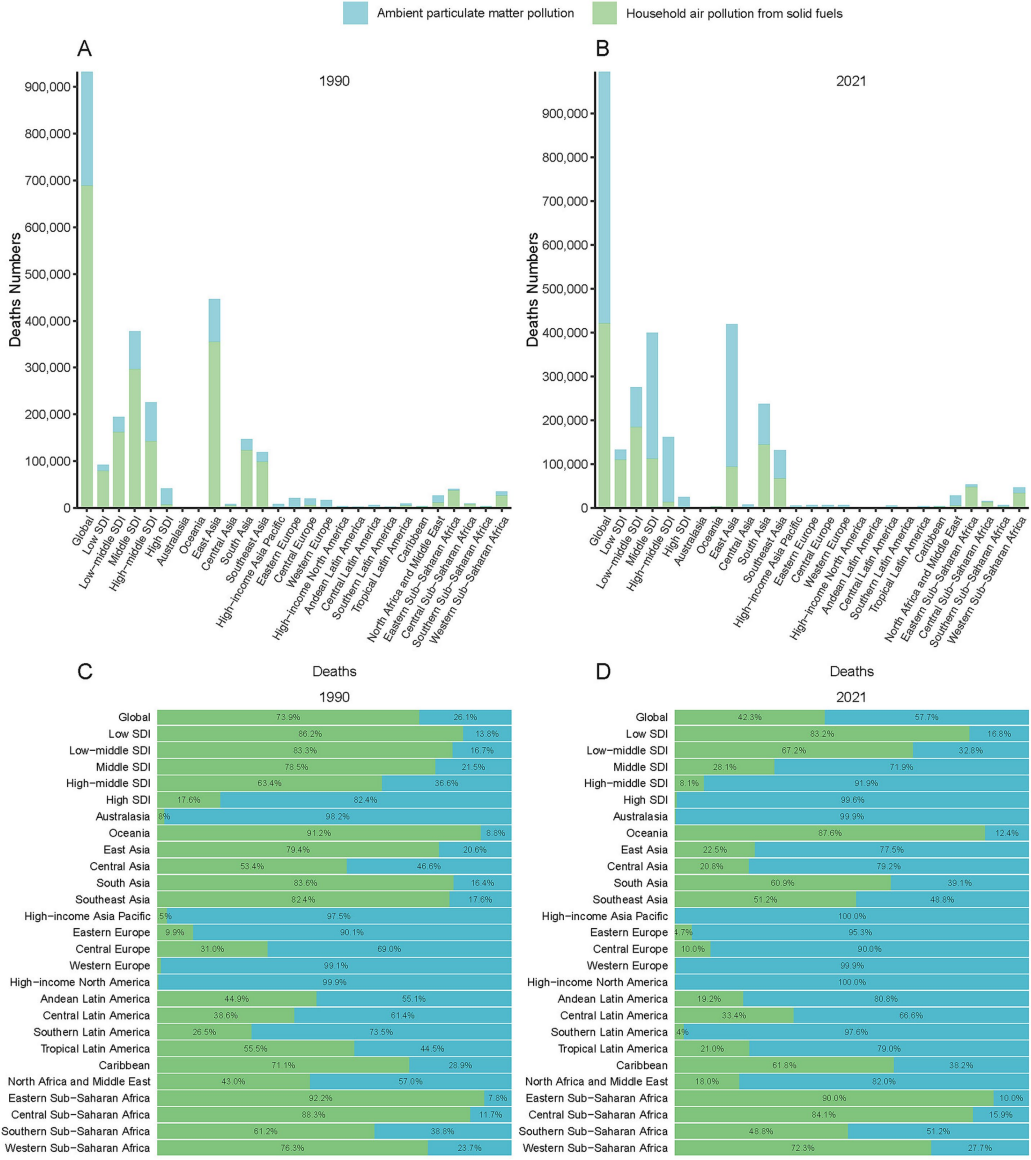
Geographic distribution of PM_2.5_-attributable ICH deaths by Global Burden of Disease (GBD) regions and SDI quintiles in 1990 **(A)** and 2021 **(B)**. Proportional contributions of pollution sources (ambient particulate matter pollution [APMP], household air pollution [HAP], and total PM_2.5_) are shown for 1990 **(C)** and 2021 **(D)**.

Globally, PM_2.5_ cause 995,650 ICH related deaths and 24,015,340 DALYs in 2021. From 1990 to 2021, ASMR and disability rates (DALYs, YLDs, YLLs) for ICH attributable to PM_2.5_ exposure showed consistent downward trends, with the AAPC values being −2.48 for ASMR, −2.52 for DALYs, −1.74 for YLDs, and −2.54 for YLLs, respectively, (Table 1; Supplementary Tables S1–S3). ASMR and disability rates declined globally (ASMR: −52.4%, DALYs: −53.1%, YLL − 53.4%, YLD − 40.7%), despite a 6.8 and 18.6% rise in absolute deaths and YLD, respectively (Supplementary Tables S1–S3). Declines were driven by reductions in HAP, while APMP-related burdens increased gradually throughout the study period (Figure 1; Supplementary Figures S1, S2). In the last 30 years, the global AAPC in the burden of PM_2.5_ - related ICH has been on a declining trend. Specifically, the AAPC values stand at −2.48 for ASMR, −2.52 for DALYs, −1.74 for YLDs, and −2.54 for YLLs (Table 1; Supplementary Tables S1–S3). Moving forward, between 2022 and 2050, predictions suggest a rise in ASMR, ASDR, YLDs, and YLLs for ICH caused by PM_2.5_, pointing to a likely increase in the associated health burden (Figure 2).

**Table 1:** Number and age-standardized DALYs rates of intracerebral hemorrhage attributable to PM_2.5_ by geographic region, with temporal trends from 1990 to 2021.

Characteristics	Death cases 1990	ASDR 1990	Death cases 2021	ASDR 2021	AAPC
	(95% UI)	(95% UI)	(95% UI)	(95% UI)	(95% UI)
Global	932.43 (759.24–1121.4)	24.55 (19.97–29.59)	995.65 (763.17–1249.51)	11.69 (8.94–14.69)	−2.48 (−2.57 to −2.39)
Female	449.17 (363.45–554.64)	21.55 (17.43–26.62)	453.01 (348.85–567.32)	9.78 (7.53–12.24)	−2.61 (−2.7 to −2.52)
Male	483.26 (384.83–583.52)	28.33 (22.61–34.25)	542.64 (405.38–686.2)	13.98 (10.45–17.73)	−2.29 (−2.61 to −1.96)
Low SDI	92.59 (72.33–112.2)	44.56 (34.7–54.31)	132.62 (99.48–161.97)	28.4 (21.44–34.86)	−1.44 (−1.59 to −1.29)
Low-middle SDI	194.67 (152.66–233.82)	34.22 (26.71–40.97)	275.55 (208.86–335.06)	20.15 (15.26–24.65)	−1.71 (−1.8 to −1.61)
Middle SDI	377.83 (300.41–470.45)	42.35 (33.72–53.01)	399.88 (295.83–530.38)	16.12 (11.86–21.43)	−3.26 (−3.81 to −2.7)
High-middle SDI	225.23 (178.45–277.51)	24.27 (19.14–29.92)	162.2 (121.92–212.16)	8.3 (6.24–10.85)	−3.41 (−3.87 to −2.95)
High SDI	41.32 (29.47–56.51)	3.71 (2.64–5.08)	24.76 (18.43–32.15)	1.15 (0.88–1.48)	−3.69 (−3.94 to −3.45)
Australasia	0.12 (0–0.35)	0.52 (0.02–1.52)	0.2 (0.12–0.3)	0.33 (0.19–0.5)	−1.09 (−1.88 to −0.29)
Oceania	1.64 (1.19–2.15)	64.52 (47.36–83.47)	3.05 (2.14–4.06)	46.53 (32.81–61.66)	−1.06 (−1.16 to −0.95)
East Asia	446.14 (346.55–566.46)	65.63 (50.97–83.3)	418.94 (302.28–555.41)	20.85 (15.1–27.69)	−3.68 (−3.95 to −3.41)
Central Asia	8.57 (5.03–12.26)	19.42 (11.4–27.78)	7.54 (5.6–9.91)	10.26 (7.62–13.45)	−2.1 (−2.77 to −1.42)
South Asia	147.02 (111.9–181.18)	27.48 (20.68–34.03)	237.68 (174.73–300.29)	16.8 (12.27–21.29)	−1.62 (−2.04 to −1.2)
Southeast Asia	119.19 (93.81–142.39)	50.81 (39.48–61.1)	131.57 (90.75–177.23)	21.26 (14.72–28.75)	−2.83 (−2.94 to −2.71)
High-income Asia Pacific	8 (2.65–15.43)	4.14 (1.36–8)	5.89 (3.41–8.83)	1.12 (0.66–1.66)	−4.07 (−4.3 to −3.84)
Eastern Europe	20.7 (10.72–32)	7.66 (3.97–11.83)	6.88 (4.27–10.41)	2 (1.25–3.03)	−4.3 (−5.27 to −3.33)
Central Europe	20.21 (11.87–28.72)	14.16 (8.33–20.12)	6.73 (4.81–9.79)	2.93 (2.1–4.26)	−5.02 (−5.36 to −4.68)
Western Europe	16.36 (8.01–27.43)	2.75 (1.35–4.61)	6.21 (4.24–8.81)	0.56 (0.39–0.79)	−5.03 (−5.31 to −4.75)
High-income North America	3.69 (1.44–6.56)	1.03 (0.41–1.83)	2.02 (0.99–3.31)	0.3 (0.15–0.49)	−3.7 (−4.52 to −2.87)
Andean Latin America	2.94 (2.23–3.71)	14.59 (11.04–18.42)	1.83 (1.16–2.71)	3.11 (1.99–4.62)	−4.84 (−5.36 to −4.31)
Central Latin America	5.69 (3.83–7.8)	7.26 (4.88–9.97)	5.07 (3.44–7.17)	2.07 (1.4–2.93)	−3.97 (−4.31 to −3.63)
Southern Latin America	3.32 (1.77–5.21)	7.29 (3.88–11.48)	1.53 (0.93–2.36)	1.73 (1.05–2.66)	−4.48 (−4.75 to −4.22)
Tropical Latin America	9.48 (5.48–14.27)	10.43 (6.08–15.64)	4.49 (2.6–6.91)	1.75 (1.01–2.69)	−5.57 (−6.03 to −5.11)
Caribbean	3.79 (2.68–5.08)	14.68 (10.39–19.75)	4.5 (3.16–6.06)	8.38 (5.89–11.3)	−1.71 (−1.92 to −1.5)
North Africa and Middle East	26.23 (19.73–33.03)	17.17 (12.98–21.62)	28.5 (21.04–36.2)	6.7 (4.95–8.49)	−3.11 (−3.33 to −2.89)
Eastern Sub-Saharan Africa	40.51 (32.05–50.15)	59.41 (47.38–72.9)	53.19 (41.62–64.53)	34.86 (26.93–42.47)	−1.7 (−1.78 to −1.63)
Central Sub-Saharan Africa	9.36 (6.83–12.41)	47.36 (34.71–62.38)	15.92 (11.08–21.54)	34.04 (23.66–46.17)	−1.06 (−1.21 to −0.92)
Southern Sub-Saharan Africa	4.2 (3.06–5.37)	16.16 (11.66–20.7)	6.79 (4.98–8.82)	12.41 (9.12–16.19)	−0.84 (−1.33 to −0.34)
Western Sub-Saharan Africa	35.28 (25.96–44.94)	43.65 (31.95–56.3)	47.14 (34.14–59.62)	26.12 (18.75–33.02)	−1.64 (−1.72 to −1.57)

Low SDI regions bore the highest burden of PM_2.5_ -related ICH. The age-standardized mortality rates (ASMR: 28.4/100,000) and DALY rates (674.7/100,000) in these regions were 24.7 times and 23.1 times higher than High SDI regions (ASMR: 1.15/100,000; DALYs: 29.2/100,000) (Figures 1A,C). This disparity was also evident in disability metrics. The YLD and YLL rates in Low SDI regions (13.98/100,000; 660.72/100,000) were 6.2 times and 24.5 times higher than those in High SDI regions (Supplementary Tables S4–S7).

Over the past three decades, total PM_2.5_- and HAP-attributable ICH burdens declined across all SDI regions (Figures 1A,C). Notably, APMP-related burdens in High-middle SDI regions began decreasing after 2005, whereas Low SDI regions experienced an APMP burden peak in 2015, followed by gradual declines. High SDI regions achieved most decreasing in ASMR due to PM_2.5_ with an AAPC of - 3.69%. Similarly, in high - middle SDI regions, the ASDR due to PMP also showed a significant decrease, registering an AAPC of - 3.64%. Conversely, low SDI regions exhibited the least pronounced downward trends in both ASMR and ASDR. Specifically, the AAPCs for ASMR and ASDR in these regions were - 1.44% and - 1.5%, respectively, (Table 1; Supplementary Table S1).

### Regional and geographic disparities in the burden of PM_2.5_-attributable intracerebral hemorrhage (1990–2021)

**Figure 3:**
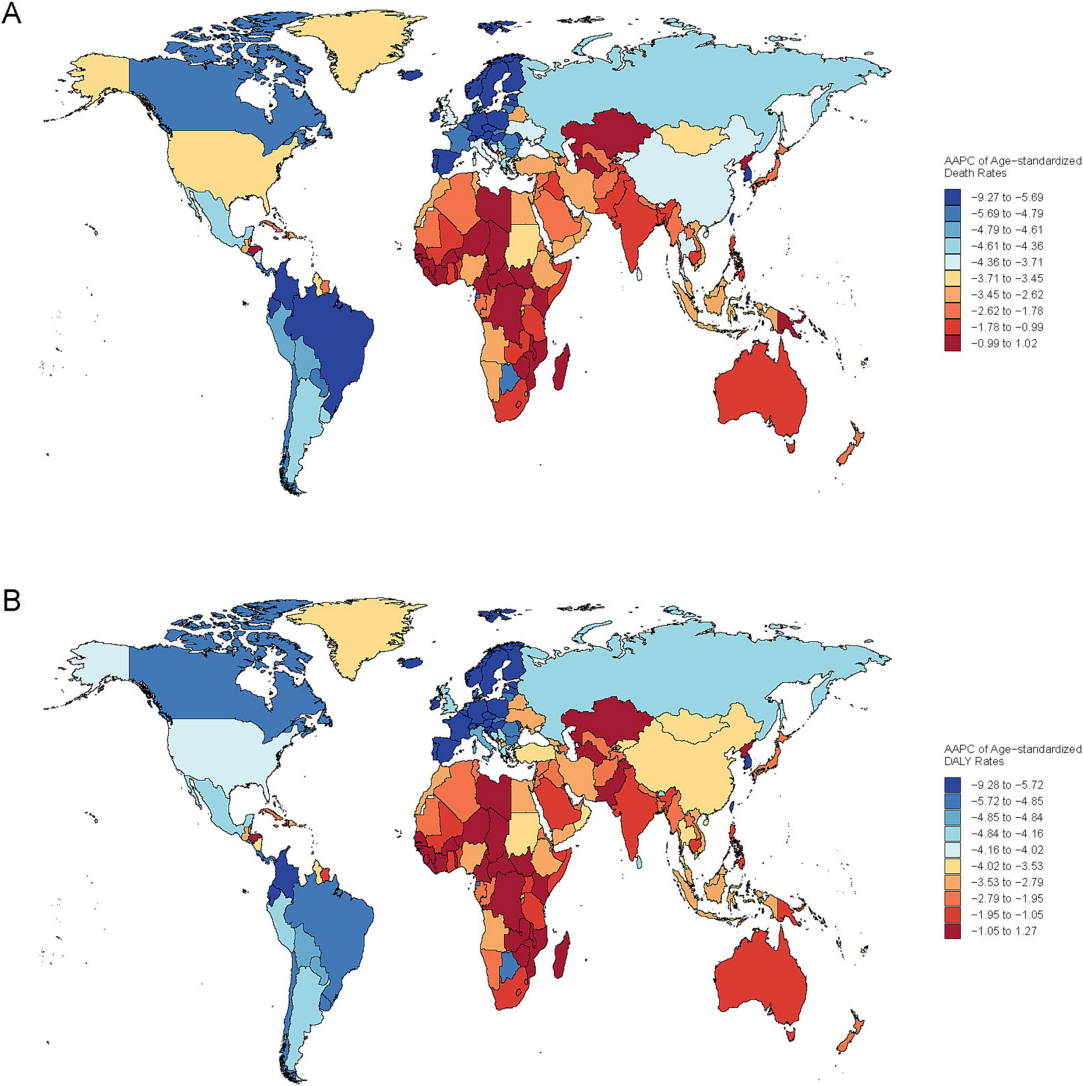
Global spatial variation in the annual average percentage change (AAPC) of age-standardized mortality rate (ASMR) **(A)** and disability-adjusted life years (DALYs) **(B)** for PM_2.5_-attributable ICH, 1990–2021.

Among GBD regions, Asian regions (East/South/Southeast Asia) had the highest death counts of ICH due to PM_2.5_. East Asia transitioned from a HAP - dominated to an APMP - dominated pollution pattern (Figures 3A,B). Western Europe, North America, and Australasia, were mainly affected by APMP, whereas Oceania, the Caribbean, and Sub - Saharan Africa, remained dominated by HAP (Figures 3A,B). Similar spatial disparities were observed for DALYs, YLLs, and YLDs throughout the study period (Supplementary Figures S4–S6). The AAPC across the 21 GBD regions showed a decreasing trend. Tropical Latin America displayed the most significant declines due to PM_2.5_, with an AAPC of −5.57% for the ASMR and −5.65% for DALYs, followed by Central and Western Europe (Table 1; Supplementary Table S1).

**Figure 4:**
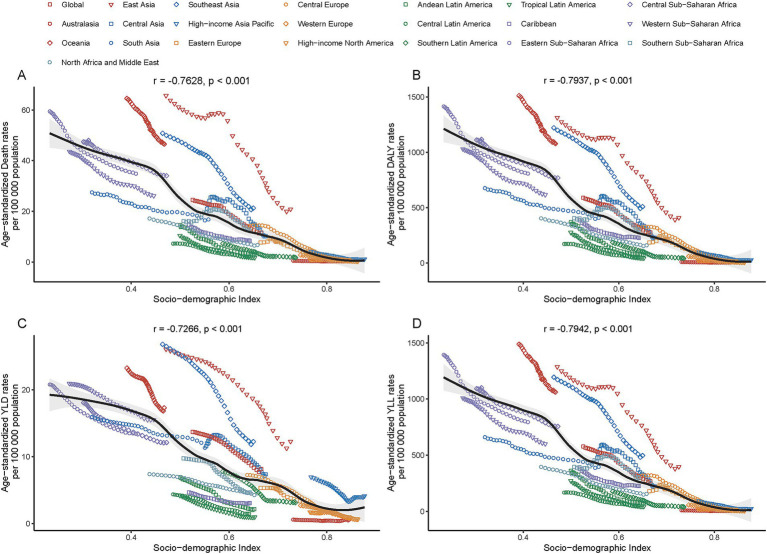
Association between age-standardized rates [deaths **(A)**, DALYs **(B)**, years lived with disability (YLDs, **C**), and years of life lost (YLLs, **D**)] of PM_2.5_-attributable ICH and the Socio-demographic Index (SDI) across 21 GBD regions in 2021.

Several countries in low and low - middle SDI regions faced high health burdens. For ASMR, Solomon Islands (77.19/100,000), Mozambique (63.38/100,000), and Madagascar (63.27/100,000) ranked the highest (Supplementary Table S4). In terms of ASDR, Laos (999.75/100,000), Malawi (991.15/100,000), and Burundi (981.62) were at the top (Supplementary Table S5). Regarding age - standardized YLDs, Solomon Islands (50.04/100,000), Vanuatu (35.70/100,000), and Kiribati (33.67/100,000) reported the highest values (Supplementary Table S6). And for age - standardized YLLs, Eritrea (999.43/100,000), Cambodia (995.88/100,000), and Kiribati (993.58/100,000) had the highest rates (Supplementary Table S7). At the national level, Estonia (Eastern Europe) exhibited pronounced reductions across ASMR, DALYs, YLDs, while the Maldives showed the most notable improvement in YLDs (Figure 4; Supplementary Figure S7).

### PM_2.5_-attributable ICH burden in 2021, SDI-stratified

**Figure 5:**
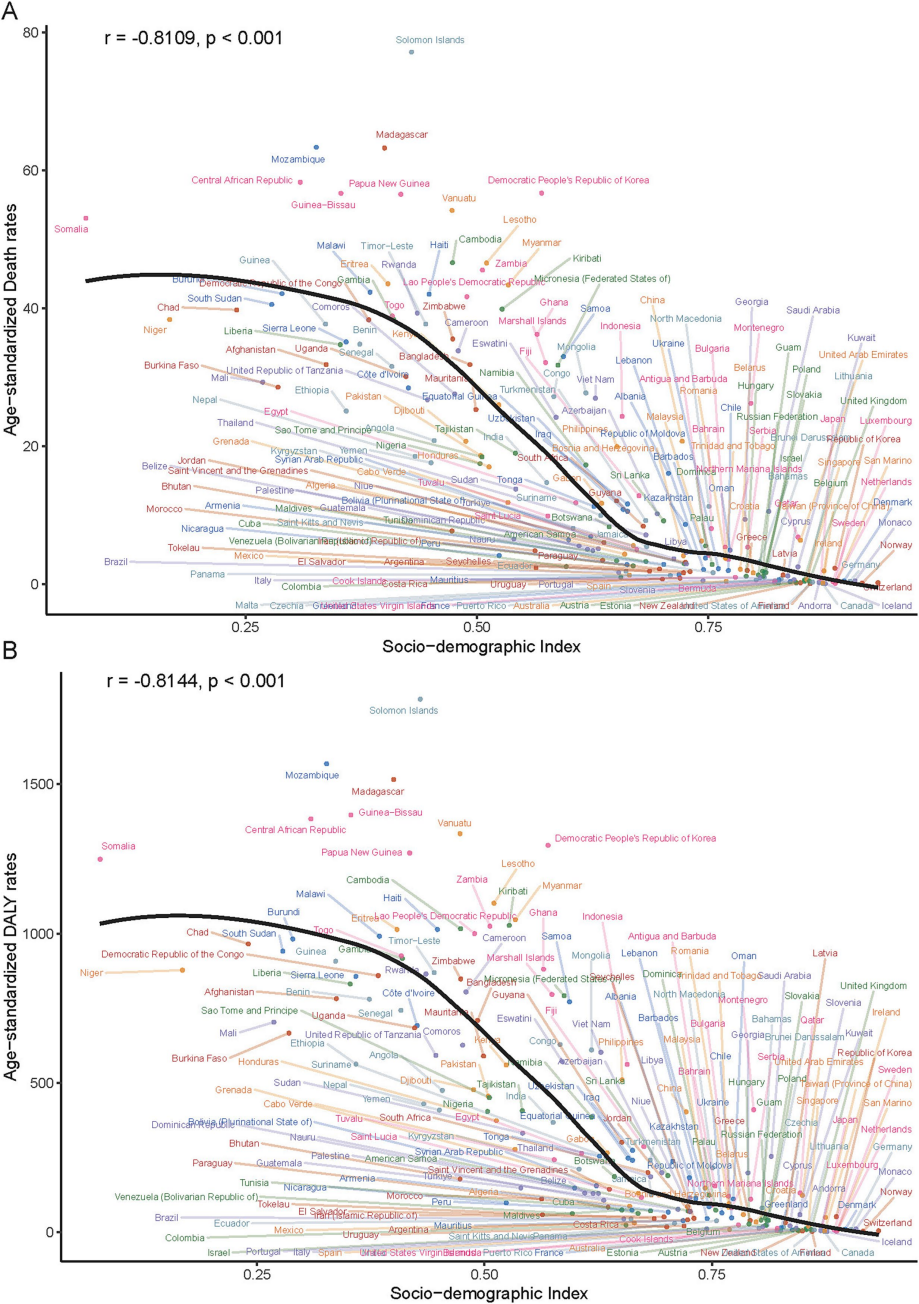
Age-standardized death **(A)** and DALY **(B)** rates of PM_2.5_-attributable ICH across 204 countries/territories stratified by SDI in 2021.

**Figure 6:**
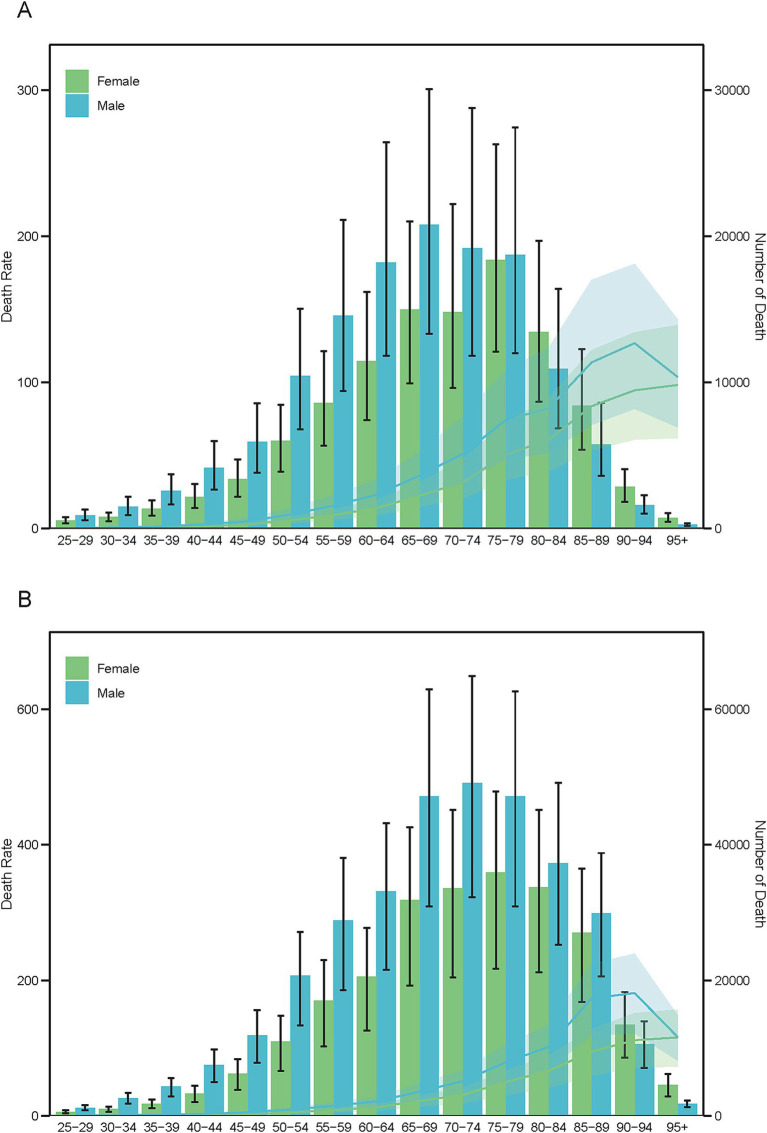
Age-specific mortality counts (bars) and age-standardized mortality rates (lines) for PM_2.5_-attributable ICH by sex in 1990 **(A)** and 2021 **(B)**.

In 2021, a significant negative correlation was observed between the SDI level and the age-standardized rates of deaths, DALYs, YLDs, and YLLs across 21 GBD regions. Specifically, the correlation coefficients and p - values were as follows: for deaths, *r* = − 0.76, *p* < 0.001; for DALYs, *r* = − 0.79, *p* < 0.001; for YLDs, *r* = − 0.73, *p* < 0.001; for YLLs, *r* = − 0.79, *p* < 0.001 (Figure 5). Additionally, a significant negative correlation was found between the age - standardized rates of ICH burden attributable to total PM_2.5_ across 204 countries and the SDI. Generally, the burden decreased as the SDI increased (*r* = − 0.81, *p* < 0.001). (Figure 6 and Supplementary Figure S8).

### Age-specific global burden of ICH due to PM_2.5_: 1990–2021

**Figure 7:**
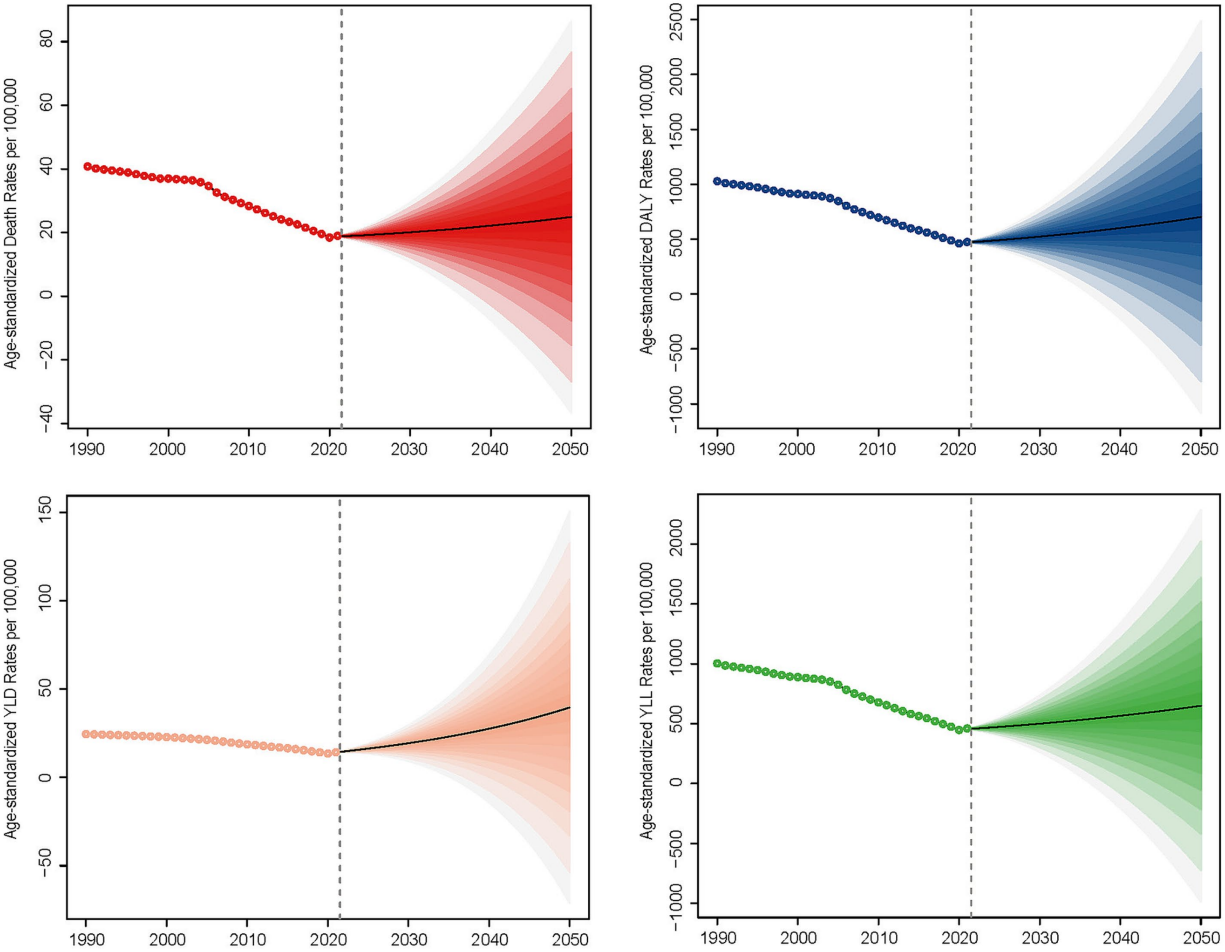
Projected trends in age-standardized mortality rates (ASMR) of PM_2.5_-attributable ICH, 2022–2050, based on quantile regression modeling.

Between 1990 and 2021, males persistently demonstrated greater disease burden from ICH associated with ambient PM_2.5_ exposure. Mortality analyses revealed notable aging trajectories across genders: Male fatalities transitioned from peaking in the 65–69 age cohort (1990) to the 70–74 group (2021), whereas female mortality consistently peaked in the 75–79 age group throughout both study periods (Figure 7). DALYs patterns evolved differentially by gender, with males experiencing a transition from peak rates at 60–64 years (1990) to 65–69 years (2021), while females maintained stable peak DALYs in the 65–69 cohort across both decades (Supplementary Figure S9). YLDs demonstrated exceptional temporal stability, with both genders sustaining maximum rates in the 55–59 age category (Supplementary Figure S10). The YLLs metric revealed distinct longitudinal patterns - males showed progressive aging of peak burden from 60–64 years (1990) to 65–69 years (2021), while females maintained consistent YLL maxima in the 65–69 age group, paralleling their DALY distribution pattern (Supplementary Figure S11).

## Discussion

The GBD 2021 analysis provides critical insights into the evolving burden of PM_2.5_-attributable ICH. Although global ASMR for ICH declined by 52.4% from 1990 to 2021, persistent disparities across socioeconomic strata highlight the dual challenges of pollution control and healthcare equity.

Current evidence identifies PM_2.5_ as a significant risk factor for both ICH and ischemic strokes, though their pathogenic mechanisms differ fundamentally. Experimental studies demonstrate that PM_2.5_ can induce the breakdown of endothelial function, resulting in increased blood–brain barrier permeability and sustained inflammatory responses, which may predispose blood vessels in the brain to rupture, thus contributing to ICH (28). In contrast, ischemic strokes primarily arise from PM_2.5_-driven atherogenesis and thrombogenesis via inflammatory arterial occlusion (29, 30). Notably, Huang et al. (31) observed that PM_2.5_ exposure triggers vasoconstriction and hypertension elevation, thereby exacerbating hemorrhagic risk. The same team quantified a 20% increased incidence of ischemic stroke (per 10 μg/m^3^; 95% CI: 1.15–1.25) versus 12% for hemorrhagic stroke (1.05–1.20) with long-term exposure (31). Conversely, Tian et al. (32) reported stronger correlations between PM_2.5_ and ischemic stroke admissions than hemorrhagic events. This aligns with studies linking short-term PM_2.5_ spikes to ischemic stroke risk (33), whereas ICH admissions show weaker associations (34, 35). The heterogeneity in these epidemiological findings may arise from several factors: (1) Differences in study populations (e.g., underlying prevalence of hypertension, a major ICH risk factor); (2) Variations in PM_2.5_ composition and sources (influencing toxicity); (3) Methodological disparities in exposure assessment windows (short-term spikes vs. long-term averages) and outcome ascertainment; and (4) The complex interplay of PM_2.5_ with other time-varying risk factors (e.g., temperature, co-pollutants) which may differentially affect stroke subtypes. Collectively, these findings underscore critical subtype-specific mechanisms and exposure-response relationships, highlighting the need for future research to dissect the contributions of specific PM components, exposure durations, and susceptible populations to resolve these apparent contradictions.

Our analysis reveals that the decline in the burdens of ICH associated with PM_2.5_ and HAP over the past three decades across different SDI regions is a manifestation of the intricate interplay among environmental, healthcare, and socioeconomic factors (36, 37). High - income countries have successfully achieved significant reductions in pollution - related cerebrovascular risks. They have accomplished this through the implementation of stringent air quality standards and comprehensive public health measures (31, 33). Regions where the Clean Air Act has been put into effect have witnessed particularly notable improvements (25). In high - middle SDI regions, coordinated policy interventions initiated since the mid - 2000s have led to tangible enhancements in both air quality and stroke outcomes, making these regions prime examples of such progress (38, 39). In Western Europe, North America, and Australasia, urbanization - driven energy demands have resulted in 100% ambient PM_2.5_ exposure. The dense populations and transportation systems heavily reliant on fossil fuels in these areas exacerbate emissions from vehicles and industries (40). Although regulatory agencies, such as the U. S. Environmental Protection Agency (EPA) and European regulators, have enforced strict pollution control measures, the continued dependence on carbon - intensive infrastructure has constrained further progress (41). Despite the gradual improvement in air quality, historical industrial emissions and ongoing traffic pollution have maintained PM_2.5_ as a critical threat to urban public health.

Low-resource communities face compounded air pollution risks from dual exposure to APMP and HAP (37, 42). Critically, lower SDI regions—constrained by limited economic resources and competing public health priorities—often deprioritize environmental governance, exacerbating these exposures. This synergistic effect leads to PM_2.5_ - attributable ICH mortality rates that are 24 times higher than those in high - income regions (43). Such a disparity reflects systemic deficiencies in pollution regulation, healthcare access, and policy enforcement (42, 44). In South Asia and Africa, heavy reliance on biomass fuels perpetuates severe indoor pollution. Simultaneously, rapid urbanization in these regions prioritizes economic growth over public health protection (40). Underfunded public health infrastructure further limits capacity to mitigate pollution-linked chronic diseases (e.g., hypertension, cerebrovascular disorders), creating a feedback loop that entrenches disease burden (45). Public misconceptions about air pollution risks further hinder household- and community-level mitigation efforts (46, 47). In African cities, air pollutant concentrations are higher in urban business and high - density residential areas due to traffic and biomass use compared to peri - urban or rural areas. Traffic - related air pollution from formal and informal transport sectors is a major health concern in urban Sub - Saharan Africa (SSA) (48, 49). In SSA cities, the number of new vehicles is rising, yet second - hand and diesel - powered vehicles are still imported. Vehicle fleets are often poorly maintained, and emissions standards are either absent or not strictly enforced in most SSA countries (50). The COVID - 19 pandemic has exacerbated these vulnerabilities. SARS - CoV - 2 infection may aggravate PM_2.5_ - related cerebrovascular damage, disproportionately affecting marginalized groups (39, 51). This interaction emphasizes the necessity of pandemic - responsive environmental health strategies that can address both acute infectious threats and chronic pollution exposure. China’s experience illustrates how targeted emission reductions yield measurable benefits, especially among pollution-sensitive older adult populations (31, 33), supported by parallel healthcare improvements (38). Nevertheless, many low - income nations only started to curb PM_2.5_ - related ICH burdens after 2015, trailing High - SDI regions by several decades (38, 43)**—**a delay attributable to economic barriers limiting early adoption of environmental health interventions.

Gender and age dynamics present crucial considerations, with males demonstrating a higher disease burden attributed to PM_2.5_ exposure across all age groups. Males generally exhibit higher incidence rates of cardiovascular diseases, and the progression of risk factors like hypertension and lifestyle diseases may contribute to an earlier peak in mortality. Studies have indicated that the impact of PM_10_ on cardiovascular-related deaths is particularly significant among men, individuals who smoke, and those with higher socioeconomic standing (52). Therefore, these factors contribute to a greater disease burden among males in the context of ICH. The noted shift in the age cohort for male fatalities—from 65–69 years in 1990 to 70–74 in 2021—implies a progressive aging of the ICH burden within this population. This trend aligns with the global demographic shift towards older populations, where increased longevity may mean older adults are living longer with comorbidities that elevate their risk for prevalent diseases such as ICH (53). The remarkably stable rates of YLDs across both genders suggest a chronic burden of morbidity associated with ICH, where recovery may not always lead to restored function or quality of life. The consistent peak of YLDs between the ages of 55–59 for both genders implies that early - life exposures or risk behaviors could predispose individuals to the long - term sequelae of ICH caused by PM_2.5_ exposure in later life. Given the pronounced gender differences, health policies must consider sex-specific approaches to manage risk factors effectively. Additionally, the intersection of aging demographics and environmental health poses significant challenges; as populations continue to age, the burden of PM_2.5_-related health issues necessitates proactive monitoring and comprehensive intervention strategies aimed at reducing exposure to air pollutants.

Longitudinal projections (2022–2050) indicate a rise in disease burden for PM_2.5_-attributable ICH. This trend underscores a critical limitation in current Sustainable Development Goal (SDG) monitoring frameworks, which fail to account for the compounding effects of population aging on absolute disease burden. Three key drivers necessitate urgent policy attention: Demographic transition: global aging populations amplify absolute ICH cases even with stable exposure levels, as evidenced by sensitivity analyses in the GBD 2021 (52, 54). Urbanization-environment nexus: rapid urban expansion in low- and middle-income countries (LMICs) exacerbates PM_2.5_ exposure while straining healthcare capacity—a dual challenge requiring spatially explicit interventions. Health inequity gradient: Socioeconomic disparities mediate PM_2.5_-ICH associations through differential access to preventive care and hypertension management, creating preventable mortality hotspots.

Our study has some limitations. Firstly, ambient air contains not only PM_2.5_ but also other pollutants such as PM₁₀, NO₂, O₃, and SO₂. These pollutants can individually or jointly influence the disease burden of ICH. Despite the progress made by the GBD 2021 in integrating pollutant data, the synergistic effects of multiple pollutants remain an uncharted area in disease burden assessment. Secondly, when conducting international comparisons of disease burden, one must exercise caution. Disparities in the quality of mortality and DALY data exist among different countries. It is extremely challenging to precisely quantify and eliminate measurement errors in epidemiological indicators, which results in data inconsistencies. In low - income countries, the data quality is a significant concern. Moreover, there is a shortage of population - representative studies for disease indicators. As a result, it becomes necessary to rely on generalized assumptions, particularly when studying stroke.

In summary, this study highlights critical trends and disparities in the global burden of PM_2.5_-attributable ICH from 1990 to 2050. While age-standardized mortality and disability rates have declined globally since 1990—driven by reductions in household and solid fuel pollution (HAP/ total PM_2.5_)—persisting inequities reveal a 24 times higher burden in Low-SDI regions compared to High SDI settings. These disparities stem from divergent pollution sources: industrialized regions face APMP from urbanization and fossil fuels, whereas low-resource areas remain entrenched in HAP due to biomass reliance. Alarmingly, projections indicate a resurgence in age-standardized rates by 2050, exacerbated by aging populations and gendered exposure risks, with males and older adults disproportionately affected.

The significance of these findings lies in their urgency for equitable policy action. To mitigate future burdens, interventions must prioritize: (1) Low SDI regions: Accelerating clean energy transitions to curb household biomass use, paired with healthcare strengthening to address lagging disease surveillance. (2) High SDI regions: Stricter emission controls on transportation and industry to combat APMP. (3) Global collaboration: Education campaigns to rectify public misconceptions and gender/age-sensitive strategies targeting high-risk groups. Without addressing these systemic gaps, PM_2.5_ pollution will perpetuate cycles of health inequality, underscoring that environmental justice is inseparable from cerebrovascular health equity.

## Data Availability

The datasets presented in this study can be found in online repositories. The names of the repository/repositories and accession number(s) can be found at: (http://ghdx.healthdata.org/gbd-results-tool.

## Footnotes

^1^ http://ghdx.healthdata.org/gbd-results-tool
