# Supplementary material for: Ambient versus household PM2.5 exposure and socioeconomic disparities in intracerebral hemorrhage burden: a 32-year global analysis (1990–2021) with projections to 2050
Source: Front Public Health. 2025 Jun 18;13:1615934. doi: 10.3389/fpubh.2025.1615934 (PMC12213839; doi:10.3389/fpubh.2025.1615934)
Supplement: Supplementary file 1 [file Data_Sheet_1.docx]

Fig. S1. Temporal trends in DALYs of PM_2.5_-attributable ICH by GBD regions and SDI quintiles, 1990–2021. Total PM_2.5_ (A), HAP(B), APMP(C).

Fig. S2 Temporal trends in YLDs of PM2.5-attributable ICH by GBD regions and SDI quintiles, 1990–2021. Total PM_2.5_ (A), HAP(B), APMP(C).

Fig. S3 Temporal trends in YLLs of PM2.5-attributable ICH by GBD regions and SDI quintiles, 1990–2021. Total PM_2.5_ (A), HAP(B), APMP(C).

Fig. S4 Proportional distribution of pollution sources (APMP, HAP, total PM_2.5_) for DALYs of PM2.5-attributable ICH by GBD regions in 1990 and 2021.

Fig. S5 Proportional distribution of pollution sources (APMP, HAP, total PM_2.5_) for YLDs of PM2.5-attributable ICH by GBD regions in 1990 and 2021.

Fig. S6 Proportional distribution of pollution sources (APMP, HAP, total PM_2.5_) for YLLs of PM2.5-attributable ICH by GBD regions in 1990 and 2021.

Fig. S7 Global AAPC trends in age-standardized YLDs (A) and YLLs (B) for PM2.5-attributable ICH, 1990–2021.

Fig. S8 Age-standardized YLD (A) and YLL (B) rates of PM_2.5_-attributable ICH across 204 countries/territories by SDI in 2021.

Fig. S9 Age-specific DALYs of PM_2.5_-attributable ICH by sex in 1990 (A) and 2021 (B).

Fig. S10
Age-specific YLDs of PM_2.5_-attributable ICH by sex in 1990 (A) and 2021 (B).

Fig. S11 Age-specific YLLs of PM_2.5_-attributable ICH by sex in 1990 (A) and 2021 (B).
